# Barriers to Surveillance for Hepatocellular Carcinoma in a Multicenter Cohort

**DOI:** 10.1001/jamanetworkopen.2022.23504

**Published:** 2022-07-01

**Authors:** Neehar D. Parikh, Nabihah Tayob, Taim Al-Jarrah, Jennifer Kramer, Jennifer Melcher, Donna Smith, Patrick Marquardt, Po-Hong Liu, Runlong Tang, Fasiha Kanwal, Amit G. Singal

**Affiliations:** Division of Gastroenterology and Hepatology, University of Michigan, Ann Arbor; Department of Biostatistics, Dana Farber Cancer Center, Boston, Massachusetts; Division of Gastroenterology and Hepatology, University of Michigan, Ann Arbor; Center for Innovations in Quality, Effectiveness and Safety, Michael E. DeBakey Veterans Affairs Medical Center, Houston, Texas; Department of Medicine, Michael E. DeBakey Veterans Affairs Medical Center and Baylor College of Medicine, Houston, Texas; Center for Innovations in Quality, Effectiveness and Safety, Michael E. DeBakey Veterans Affairs Medical Center, Houston, Texas; Department of Medicine, Michael E. DeBakey Veterans Affairs Medical Center and Baylor College of Medicine, Houston, Texas; Center for Innovations in Quality, Effectiveness and Safety, Michael E. DeBakey Veterans Affairs Medical Center, Houston, Texas; Department of Medicine, Michael E. DeBakey Veterans Affairs Medical Center and Baylor College of Medicine, Houston, Texas; Department of Internal Medicine, University of Texas Southwestern Medical Center, Dallas; Department of Internal Medicine, University of Texas Southwestern Medical Center, Dallas; Fred Hutchinson Cancer Research Center, Seattle, Washington; Center for Innovations in Quality, Effectiveness and Safety, Michael E. DeBakey Veterans Affairs Medical Center, Houston, Texas; Department of Medicine, Michael E. DeBakey Veterans Affairs Medical Center and Baylor College of Medicine, Houston, Texas; Division of Gastroenterology and Hepatology, Department of Medicine, Baylor College of Medicine, Houston, Texas; Department of Internal Medicine, University of Texas Southwestern Medical Center, Dallas; Harold C. Simmons Cancer Center, University of Texas Southwestern Medical Center, Dallas

## Abstract

**IMPORTANCE:**

Hepatocellular carcinoma (HCC) surveillance is underused in clinical practice, which may be owing to patient and clinician barriers.

**OBJECTIVE:**

To characterize HCC surveillance barriers and associations with clinical outcomes in a multicenter cohort of patients with cirrhosis.

**DESIGN, SETTING, AND PARTICIPANTS:**

This retrospective, multicenter cohort study included 5 medical centers in the United States. Patients with cirrhosis and newly diagnosed HCC treated from 2014 to 2018 were included. Data were analyzed from June 2021 to February 2022.

**EXPOSURE:**

Surveillance completion in the 36-month period prior to HCC diagnosis.

**MAIN OUTCOMES AND MEASURES:**

Surveillance receipt was classified as semiannual, annual, or no surveillance. Multivariable logistic regression analysis was used to identify factors associated with semiannual surveillance. We conducted multivariable logistic and Cox regression analyses to characterize associations between surveillance completion with curative treatment and overall survival.

**RESULTS:**

A total 629 eligible patients (median [IQR] age, 63.6 [56.2-71.0] years; 491 [78.1%] men) were assessed, including 7 American Indian or Alaska Native patients (1.1%), 14 Asian patients (2.2), 176 Black patients (28.0%), 86 Hispanic patients (13.1%), and 340 White patients (54.1%). Nearly two-thirds of the cohort had no surveillance prior to HCC diagnosis (mean [range by site] 63.7% [37.9%-80.4%]), with a mean (range by site) of 14.0% (5.3%-33.3%) of patients having received semiannual surveillance and 22.3% (14.3%-28.8%) of patients having received annual surveillance. The most common reasons for no surveillance were lack of surveillance orders or nonadherence (mean [range by site], 82.4% [66.7%-92.4%], although a mean (range by site) of 17.6% (10.2%-22.1%) of patients had unrecognized cirrhosis at HCC presentation. Semiannual surveillance was associated with hepatitis B infection (odds ratio [OR], 3.06 [95% CI, 1.24-7.23]) and inversely associated with Black race (OR, 0.41 [95% CI, 0.20-0.80]) and lack of cirrhosis recognition (OR, 0.14 [95% CI, 0.02-0.46]). Semiannual HCC surveillance was significantly associated with curative treatment receipt (OR, 2.73 [95% CI, 1.60-4.70]) but not overall survival (HR, 0.81 [95% CI, 0.55-1.18]).

**CONCLUSIONS AND RELEVANCE:**

In this cohort study of patients with cirrhosis, HCC surveillance was underused in more than 80% of patients and associated with failures across the screening process. Dedicated programs to improve cirrhosis detection and HCC surveillance attainment are needed.

## Introduction

Hepatocellular carcinoma (HCC) is the third leading cause of cancer-related death worldwide, with an increasing mortality in many countries.^1^ Poor HCC prognosis is, in part, attributed to surveillance underuse in patients with cirrhosis, leading to a large proportion of HCC being detected beyond an early stage.^2-4^ HCC surveillance with biannual abdominal ultrasonography is associated with early tumor detection, curative treatment receipt, and overall survival (OS).^2^ However, fewer than one-quarter of patients with cirrhosis who are at risk undergo HCC surveillance.^5^ HCC surveillance completion is even lower among racial and ethnic minority groups, such as Black and Hispanic patients and patients with low socioeconomic status, contributing to disparities in HCC outcomes.^6,7^

Effective cancer screening requires several steps along a continuum of care, including recognition of patients who are at risk, clinicians recommending and ordering screening, and patients adhering to complete surveillance tests in a timely manner.^8,9^ Failures in surveillance can occur anywhere along the continuum related to patient-, clinician-, and system-level factors.^10^ Prior studies evaluating barriers to HCC surveillance have suggested patient-level factors, including logistical barriers (eg, transportation), and clinician-level factors, including knowledge and clinic time constraints, are associated with HCC surveillance receipt.^11-13^ These barriers may be particularly prevalent for racial and ethnic minority groups, contributing to observed disparities in HCC prognosis.^14^ However, most existing studies are single-center analyses, and it is unclear if observed barriers are consistent across different settings and health systems, highlighting a need for multicenter studies with large, diverse cohorts. We aimed to perform a multicenter retrospective cohort study to characterize barriers to HCC surveillance in 5 distinct medical centers representing different health care settings in the US.

## Methods

This cohort study was approved by the institutional review boards at all sites, with data use agreements for data sharing between sites. Given the retrospective nature of the study, a waiver of informed consent was granted by the institutional review boards at each site. We followed the Strengthening the Reporting of Observational Studies in Epidemiology (STROBE) reporting guideline for cohort studies.

### Patient Selection

We included patients with cirrhosis who were diagnosed with HCC between January 2014 and December 2018 in 5 health systems in the US, including 3 tertiary care referral centers, a safety-net center, and a Veterans Health Administration hospital. At each site, patients with HCC were identified by *International Classification of Diseases, Ninth Revision* (*ICD-9*) and *International Statistical Classification of Diseases and Related Health Problems, Tenth Revision* (*ICD-10*) codes and tumor board presentation lists. HCC diagnoses were confirmed using American Association of Liver Disease criteria: histological findings confirming HCC or imaging with a Liver Imaging Reporting and Data System–5 lesion that is at least 1 cm.^15^ All patients were required to have an outpatient primary care or hepatology clinic visit encounter at the site at least 12 months prior to HCC diagnosis, allowing for surveillance failures to be accurately characterized. Patients without cirrhosis and those with Child-Pugh C cirrhosis were excluded because surveillance is not recommended in those subgroups. In the absence of overt hepatic decompensation, liver histological findings, or F4 fibrosis detected on transient or magnetic resonance elastography, a diagnosis of cirrhosis was based on a combination of radiological and laboratory test results in which patients must have met 2 of the following 4 criteria: radiological imaging showing features of cirrhosis (nodular liver, intra-abdominal collaterals, varices, or splenomegaly), platelet count fewer than 100×10^3^ cells/μL (to convert to ×10^9^ cells/L, multiply by 1), endoscopy showing esophageal or gastric varices, or Fibrosis-4 Index (FIB-4) greater than 3.25.

### Surveillance Exposure

We categorized patients based on receipt of HCC surveillance, type of surveillance testing (abdominal ultrasonography, abdominal ultrasonography and α-fetoprotein, abdominal contrast-enhanced magnetic resonance imaging [MRI] or computed tomography [CT]) and whether their HCC was detected by a surveillance examination, based on the clinical notes associated with the imaging examination that detected the HCC. Surveillance was assessed from the time of first primary care or hepatology encounter prior to HCC diagnosis, with a maximum period of 36 months prior to HCC diagnosis. Specifically, a patient who was first seen more than 3 years prior to HCC diagnosis had surveillance completion assessed over the entire 36-month surveillance study period, whereas a patient who had their first clinic visit 1 year prior had surveillance completion assessed over the 12-month period. Surveillance completion was classified into 3 mutually exclusive categories. Semiannual surveillance was defined as abdominal ultrasonography, contrast-enhanced CT, or contrast-enhanced MRI a mean (SD) of every 6 (1) months during the surveillance study period prior to HCC diagnosis. Annual surveillance was defined as at least 1 abdominal imaging study annually during the surveillance study period without meeting criteria for semiannual surveillance. Patients with annual abdominal imaging performed less than annually during the surveillance study period were classified as no surveillance. We conducted a secondary analysis using proportion time covered (PTC), with each abdominal imaging examination considered to be providing 6 months of surveillance coverage, divided by months seen at a health system within a maximum of 36 months prior to HCC diagnosis.^16^ For both analyses, inclusion of imaging examinations was agnostic to intent of the imaging examination, so diagnostic and surveillance imaging were both counted toward surveillance categories and PTC calculations.

We classified reasons for surveillance failure into 2 mutually exclusive categories: (1) failure to recognize cirrhosis, and (2) failure of clinicians to order surveillance or patients to complete surveillance, among those with recognized cirrhosis. Failure to recognize cirrhosis was defined as lack of any specific testing (eg, viral hepatitis serologies) or mention of liver disease or cirrhosis in clinical notes. Failure to order or complete surveillance was defined as lack of surveillance testing results or documentation of surveillance receipt in clinical notes.

### Clinical Outcomes of Interest

Common data elements were developed, with centralized entry of data from each site using electronic forms, and all data were extracted from the medical records by trained research coordinators. We collected data on stage of HCC at diagnosis, treatment received, and OS. Early stage was defined as Barcelona Clinic Liver Cancer (BCLC) stage 0 or A. Curative treatments were defined as local ablation, surgical resection, or liver transplantation. OS from time of HCC diagnosis was obtained from the electronic medical record and cancer registries at each site.

### Statistical Analysis

We estimated the proportions of patients receiving semiannual, annual, and no surveillance overall and within each site. We assessed patient sociodemographic and clinical characteristics, including patient age at HCC diagnosis, sex, race and ethnicity, insurance status, body mass index (BMI; calculated as weight in kilograms divided by height in meters squared), etiology of liver disease, Child-Pugh class, recognition of cirrhosis prior to HCC diagnosis, and study site as independent variables. Race and ethnicity were self-reported. Multivariate logistic regression was performed to identify factors associated with semiannual surveillance. We also examined the association of surveillance receipt with clinical outcomes, including early-stage HCC detection and curative treatment receipt using multivariable logistic regression and OS using Cox regression analysis. Multivariable models were adjusted for sociodemographic and clinical characteristics and ECOG performance status (curative treatment and survival models only). Our primary models did not adjust for site, given the risk of obscuring demographic factors associated with outcomes with site adjustment; however, all models were also conducted with site adjustment in sensitivity analysis.^17^

*P* values were 2-sided, and statistical significance was set at *P* < .05. Analyses were conducted using R statistical software version 3.6.2 (R Project for Statistical Computing). Data were analyzed from June 2021 to February 2022.

## Results

### Patient Characteristics

Of 629 patients who met inclusion criteria, the median (IQR) age was 63.6 (56.2%-71.0%) years, and 491 patients (78.1%) were men (Table 1). The cohort was diverse regarding race and ethnicity, with 7 American Indian or Alaska Native patients (1.1%), 14 Asian patients (2.2), 176 Black patients (28.0%), 86 Hispanic patients (13.1%), and 340 White patients (54.1%), and insurance coverage, including 297 patients (47.2%) with Medicare, 159 patients (25.3%) with commercial or private insurance, 67 patients (10.7%) with Medicaid, and 28 patients (4.5%) who were uninsured. The most common primary etiologies of liver disease were hepatitis C infection (422 patients [67.1%]), followed by alcohol (83 patients [13.2%]), nonalcohol-associated fatty liver disease (67 patients [10.7%]), and hepatitis B infection (27 patients [4.3%]) (Table 1).

Most patients had early-stage HCC (BCLC 0/A: 409 patients [65.0%]); however, one-fifth of the cohort was diagnosed at an advanced or terminal stage (BCLC C/D: 126 patients [20.0%]). The most common initial treatment for HCC was transarterial chemoembolization (266 patients [42.3%]), followed by local ablation (96 patients [15.3%]), stereotactic body radiation therapy (71 patients [11.3%]), and surgical resection (63 patients [10.0%]). A small proportion of patients received systemic therapy (39 patients [6.2%]) as initial treatment, while 72 patients (11.4%) received no HCC therapy.

### Surveillance Attainment

Over a median (IQR) of 36 (25-36) months prior to HCC diagnosis, a mean (range by site) of 14.0% (5.3%-33.3%) of patients received semiannual surveillance and 22.3% (14.3%-28.8%) of patients received annual surveillance. The mean (SD) PTC by surveillance was 49.7% (30.8%) (Figure). The most common imaging modality for HCC surveillance was ultrasonography (mean [SD], 60.9% [38.2%]), followed by contrast MRI (mean [SD], 21.6% [33.1%]), and contrast CT (mean [SD], 17.3% [27.9%]). Three-fourths of patients with HCC were detected by surveillance imaging (474 patients [75.4%]), although a significant proportion of these patients did not receive semiannual or annual surveillance. HCC was detected incidentally in 64 patients (10.2%) of patients, while 39 patients (6.2%) presented with symptomatic HCC, and 27 patients (4.3%) with isolated elevated α-fetoprotein.

Surveillance underuse was attributed to multiple failures, including a mean (range by site) of 17.6% (10.2%-22.1%) of patients having unrecognized cirrhosis prior to HCC diagnosis and 446 patients (82.4%) having lack of surveillance orders by clinicians or patients failing to complete surveillance after ordered.

In multivariable analysis, semiannual surveillance was significantly associated with hepatitis B infection (vs hepatitis C: odds ratio [OR], 3.06 [95% CI, 1.24-7.23]) and negatively associated with Black race (vs White race: OR, 0.41 [95% CI, 0.20-0.80]) and lack of cirrhosis recognition prior to HCC diagnosis (OR, 0.14 [95% CI, 0.02-0.46]) (Table 2). In a multivariable model including site, there were significant site-level differences and consistent associations with hepatitis B liver disease etiology and lack of cirrhosis recognition; however, Black race was no longer associated with decreased likelihood of semiannual surveillance (eTable 1 in the Supplement).

### Association Between HCC Surveillance and Clinical Outcomes

Patients underwent curative treatment in 38 patients (43.2%) in the semiannual surveillance group, compared with 23 patients (16.4%) in the annual group and 105 patients (26.2%) of the no surveillance group. In multivariable analysis, semiannual surveillance was significantly associated with receipt of curative treatment (hazard ratio [HR], 2.73 [95% CI, 1.60-4.70]) (Table 3). However, semiannual surveillance was no longer associated with curative treatment receipt after adjusting for site (HR, 1.73 [95% CI, 0.96-3.10]) (eTable 2 in the Supplement). In secondary analysis, PTC by surveillance was also associated with curative treatment receipt (OR, 1.10 [95% CI, 1.03-1.18]) (eTable 3 in the Supplement) but was also no longer associated after adjusting for site (OR, 1.03 [95% CI, 0.95-1.11]) (eTable 4 in the Supplement).

Over a median (IQR) follow-up of 31.7 (2.6-59.5) months after HCC diagnosis, 279 patients (44.4%), 288 patients (45.8%) were deceased, and 62 patients (9.9%) were lost to follow-up. In multivariable analysis, semiannual and annual surveillance were not associated with OS (Table 3), which was consistent in site-adjusted models (eTable 2 in the Supplement). PTC by surveillance was also not associated with OS (eTable 3 in the Supplement).

In a post hoc analysis, patients with HCC detected by a surveillance examination had significantly higher early-stage HCC detection (OR, 2.56 [95% CI, 1.67-3.93]) and curative treatment receipt (OR, 2.03 [95% CI, 1.21-3.51]), as well as improved survival (HR, 0.69 [95% CI, 0.52-0.91]) compared with those who were diagnosed symptomatically or incidentally. In site-adjusted models, results were similar (early detection: OR, 2.67 [95% CI, 1.72-4.16]; curative treatment: OR, 1.48 [95% CI, 0.85-2.65]; OS: HR, 0.69 [95% CI, 0.51-0.92])

## Discussion

In this multicenter cohort study of patients with cirrhosis from 5 centers in the US, we found underuse of HCC surveillance with only 1 in 7 patients receiving semiannual surveillance and 1 in 4 patients receiving annual surveillance. Although this was most often associated with clinicians failing to order surveillance or patient nonadherence with clinician orders, we noted that nearly one-fourth of surveillance underuse could be attributed to lack of cirrhosis recognition prior to HCC presentation. Semiannual surveillance and proportion time covered by surveillance were associated with receipt of curative treatment but not OS in multivariable models.

Prior studies that have examined system-level barriers to HCC surveillance have predominantly been single-center studies or analyses of administrative data sets without granular data. In this multicenter study, we found low overall surveillance attainment, with only 36.3% of patients having semiannual or annual surveillance over the study period. While this is higher than reported in a 2021 meta-analysis,^5^ significant gaps remain in surveillance attainment. Possible methods to improve surveillance include patient-centered methods, such as patient navigation or mailed outreach,^18^ or clinician-centered methods, such as best-practice advisories embedded within the electronic health record.^19^ We observed significant site-level variation in surveillance attainment, highlighting particular opportunities to target interventions at low-performing sites or patients at high risk of surveillance failure. A proof-of-concept analysis suggested that this strategy would be feasible and cost-effective.^20,21^ Furthermore, as evidence builds for biomarker-based surveillance, with several maturing phase III biomarker cohorts,^22-24^ there may be improved surveillance adherence with increased patient acceptance and removal of logistical barriers to surveillance completion.^25^

Routine measurement and reporting of surveillance receipt as a quality metric for hepatology care has been proposed; however, our study highlights a couple of points to consider. The first question is regarding the optimal metric to consider and if this should be semiannual surveillance, PTC, or proportion with surveillance-detected HCC. We found discordance in these metrics in this study, exemplified by the fact that most patients were detected by surveillance despite intermittent use not meeting the definition for annual surveillance. Our data highlight that this last metric may be the most clinically relevant, given its association with downstream outcomes, including OS, albeit potentially more difficult to measure in practice. The second is the appropriate population in which to measure this metric, given that HCC is frequently diagnosed in patients with unrecognized cirrhosis. We found 15.4% of patients did not have known cirrhosis prior to the diagnosis of HCC, which is consistent with prior studies showing up to 25% of patients had undiagnosed cirrhosis at the time of HCC diagnosis.^26,27^ This proportion may be even higher in patients with nonalcohol-related fatty liver disease, in whom HCC is prone to develop in the absence of cirrhosis.^28^ These studies highlight the need for better identification algorithms and diagnostic pathways for patients with advanced fibrosis or cirrhosis who are at risk for HCC, as well as development of risk stratification biomarkers.^29^ Population-based screening using existing noninvasive fibrosis markers has yet to be shown to be feasible,^30^ and ongoing efforts to improve cirrhosis detection are needed.

In our study, HCC semiannual surveillance was not associated with OS, which has several possible explanations. First, all patients were required to have a primary care or hepatology visit, and high levels of linkage to care could lead to frequent nonsurveillance-related abdominal imaging or receipt of intermittent surveillance that did not meet the definition for annual or semiannual surveillance.^31^ This may explain the high proportion of patients with surveillance-detected HCC despite low proportions with semiannual surveillance. Alternatively, surveillance implementation is 1 step in a larger continuum, and patients who underwent surveillance may have still experienced downstream failures, such as diagnostic delays, therapeutic delays, or underuse of curative treatments.^32,33^ Third, this may reflect the ongoing debate regarding the effectiveness of HCC surveillance programs, particularly with some studies failing to find an association with improved survival.^34-37^ A large randomized clinical trial in China demonstrated a decrease in HCC-related mortality with surveillance among patients with chronic hepatitis B infection^38^; however, supporting evidence in patients with cirrhosis is largely limited to cohort studies, which have known limitations, including lead time bias, length time bias, and residual confounding.^39^ The lack of level-1 evidence to support surveillance-related improvements in survival and patient quality of life has fueled controversy around HCC surveillance and limited its uptake in clinical practice owing to lack of integration into national screening recommendations, outside of professional societal guidelines.^40^ Furthermore, there is a dearth of data on contemporary populations of patients, such as those with nonalcohol-related fatty liver disease and post-sustained virologic response cirrhosis, in whom indolent growth patterns may be more likely and there are higher competing risks of noncancer mortality, potentially increasing chances of overdiagnosis.^41-43^ Despite these imperfect data, several studies have demonstrated a favorable risk-benefit ratio of HCC surveillance, and modeling studies have suggested the practice is likely cost-effective.^43,44^

### Limitations

Our study has several strengths and limitations of note. First, we were limited by the retrospective nature of the data analyzed, which could be impacted by several forms of bias, including residual confounding. Second, while we searched the health records, including external records, for surveillance receipt, there is the possibility of ascertainment bias if studies were conducted outside the centers included in this study. This ascertainment bias could have also affected the clinical outcomes, including early-stage HCC detection, curative treatment receipt, and OS. Furthermore, given the retrospective nature of the study, we could not determine if the intent of all included imaging examinations were for surveillance or other clinical indications. However, examinations conducted for symptoms would still obviate the need for surveillance examinations, so these were counted toward surveillance attainment. Of note, we were able to ascertain surveillance intent of the imaging examination that detected HCC. Third, we were unable to determine if surveillance underuse was due to clinicians not ordering the surveillance examinations or patient nonadherence. Prior studies suggest that the former is the more common, with patients typically adhering with surveillance orders are present.^45^ These limitations should be considered in light of the study's notable strengths, including a large multicenter cohort from multiple practice settings around the US.

## Conclusions

In this multicenter cohort study, we found underuse of HCC surveillance, with significant site-level variation. Our findings point to several gaps in cirrhosis care and the need for interventions to improve recognition of cirrhosis, as well as more convenient, accurate surveillance tests to improve adherence.

## Supplementary Material

Supplementary Tables 1-4 **eTable 1.** Factors Associated With Adequate Surveillance, Adjusted by Site
**eTable 2.** Factors Associated With Curative-Intent Therapy of and Overall Survival, Adjusted By Site
**eTable 3.** Factors Associated With Curative-Intent Therapy and Overall Survival, With Percentage of Time Under Surveillance
**eTable 4.** Factors Associated With Curative-Intent Therapy and Overall Survival, With Percentage of Time Under Surveillance and Adjusted for Site

## Figures and Tables

**Figure.**
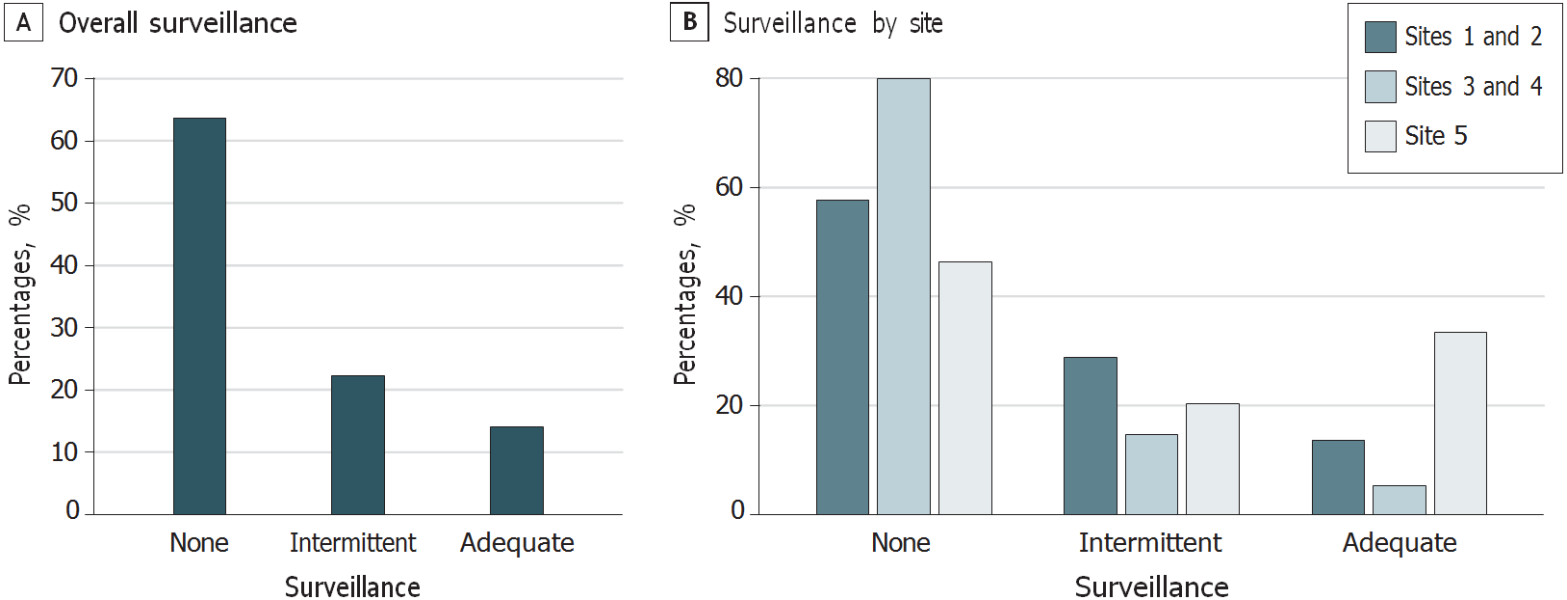
Surveillance Attainment Categories Prior to Hepatocellular Carcinoma Diagnosis for the Entire Cohort and Stratified by Site

**Table 1.** Patient Characteristics in the Overall Cohort

Overall	No. (%) (N = 629)
Age at HCC diagnosis, median (IQR), y	63.6 (56.2-71.0)
Sex	
Men	491 (78.1)
Women	138 (21.9)
Race	
American Indian or Alaska Native	7 (1.1)
Asian	14 (2.2)
Black	176 (28.0)
White	340 (54.1)
Other^a^	23 (3.7)
Unknown	69 (11.0)
Hispanic ethnicity	86 (13.7)
BMI, mean (SD)	29.3 (6.0)
Primary liver disease etiology	
Viremic HCV	321 (51.0)
Post-SVR HCV	101 (16.1)
Alcohol related liver disease	83 (13.2)
Non-alcohol associated fatty liver disease	67 (10.7)
Hepatitis B	27 (4.3)
Cryptogenic	17 (2.7)
Other	13 (2.1)
Primary health care insurance	
Medicare	297 (47.2)
Commercial or private insurance	159 (25.3)
Medicaid	67 (10.7)
Other or VA	78 (12.4)
Uninsured	28 (4.5)
Child-Pugh classification	
Class A	389 (61.8)
Class B	240 (38.2)
BCLC stage	
Stage 0 (very early stage)	68 (10.8)
Stage A (early stage)	341 (54.2)
Stage B (intermediate stage)	83 (13.2)
Stage C (advanced stage)	122 (19.4)
Stage D (terminal stage)	4 (0.6)
Unknown/Missing	11 (1.7)
Cirrhosis known prior to HCC diagnosis	
Known	532 (84.6)
Unknown	97 (15.4)

Abbreviations: BCLC, Barcelona Clinic Liver Cancer; BMI, body mass index (calculated as weight in kilograms divided by height in meters squared); HCC, hepatocellular carcinoma; HCV, hepatitis C virus; SVR, sustained virological response.

a Other race category includes Pacific Islander patients and those who identified as more than 1 race.

**Table 2:** Factors Associated With Semiannual Surveillance in Multivariable Analysis

Variable	Odds Ratios (95% CI)	*p*-value
Age at diagnosis, per 1 y	1.03 (0.99-1.06)	.13
Women (vs men)	0.94 (0.49-1.73)	.85
Race		
Black	0.41 (0.20-0.80)	.01
White	1 [Reference]	NA
Other^a^	0.83 (0.27-2.30)	.74
Hispanic ethnicity	0.40 (0.10-1.52)	.17
BMI, per 1 unit	1.04 (1.00-1.08)	.06
Etiology		
Viremic HCV infection	1 [Reference]	
Post-SVR HCV infection	1.43 (0.75-2.69)	.27
Alcohol-related liver disease	1.01 (0.45-2.15)	.98
Nonalcohol-associated fatty liver disease	1.10 (0.47-2.48)	.82
Hepatitis B virus infection	3.06 (1.24-7.23)	.01
Commercial or private insurance (vs Medicare)	0.80 (0.46-1.34)	.39
Child Pugh Class B (vs Child-Pugh class A)	1.47 (0.90-2.39)	.12
Lack of cirrhosis recognition prior to HCC diagnosis	0.14 (0.02-0.46)	.007

Abbreviations: BMI, body mass index (calculated as weight in kilograms divided by height in meters squared); HCV, hepatitis C virus; NA, not applicable; SVR, sustained virological response.

a Other race category includes American Indian or Alaska Native patients, Asian patients, Pacific Islander patients, and those who identified as more than 1 race.

**Table 3:** Factors Associated With Curative Therapy Receipt and Overall Survival in Multivariable Analysis

	Curative Intent Therapy		Overall Survival	
Variable	Odds Ratios (95% CI)	*p*-value	Hazard Ratios (95% CI)	*p*-value
Age at diagnosis, per 1 y	0.99 (0.97 – 1.02)	.71	1.01 (0.99-1.03)	.30
Women	1.31 (0.79-2.13)	.29	0.83 (0.60-1.15)	.26
Race				
Black	1.14 (0.72-1.80)	.57	1.38 (1.03-1.85)	.03
White	1 [Reference]	NA	1 [Reference]	NA
Other^a^	0.68 (0.25-1.69)	.42	0.77 (0.45-1.31)	.33
Hispanic ethnicity	0.40 (0.13-1.25)	.11	1.00 (0.55-1.83)	>.99
BMI	1.01 (0.98-1.05)	.42	0.97 (0.95-0.99)	.006
Etiology of liver disease				
Viremic HCV infection	1 [Reference]	NA	1 [Reference]	NA
Post-SVR HCV infection	0.55 (0.30-0.97)	.04	0.87 (0.57-1.34)	.52
Alcohol-related liver disease	1.01 (0.53-1.88)	.98	1.05 (0.70-1.57)	.83
Nonalcohol-associated fatty liver disease	0.97 (0.48-1.90)	.92	1.56 (1.04-2.33)	.03
Hepatitis B virus infection	0.34 (0.12-0.85)	.03	1.39 (0.84-2.32)	.20
Commercial insurance (vs Medicare)	1.06 (0.70-1.60)	.78	0.97 (0.75-1.25)	.81
ECOG propensity score				
0	1 [Reference]	NA	1 [Reference]	NA
1	1.21 (0.74-1.95)	.45	1.08 (0.80-1.47)	.62
≥2	1.29 (0.58-2.71)	.51	2.41 (1.61-3.60)	<.001
Child Pugh Class B (vs Child-Pugh A)	0.40 (0.26-0.62)	<.001	1.76 (1.37-2.27)	<.001
Semi-annual surveillance	2.73 (1.60-4.70)	<.001	0.81 (0.55-1.18)	<.001
Annual surveillance	0.62 (0.36-1.05)	.08	0.98 (0.73-1.31)	0.89

Abbreviations: BMI, body mass index (calculated as weight in kilograms divided by height in meters squared); ECOG, Eastern Cooperative Oncology Group; HCV, hepatitis C virus; NA, not applicable; SVR, sustained virological response.

a Other race category includes American Indian or Alaska Native patients, Asian patients, Pacific Islander patients, and those who identified as more than 1 race.

## Key Points

### Question

What are the factors associated with noncompletion of hepatocellular carcinoma (HCC) surveillance in patients with high risk?

### Findings

In this cohort study including 629 patients with HCC, most patients did not undergo semiannual surveillance prior to diagnosis with HCC owing to lack of clinician orders, lack of patient adherence, or lack of cirrhosis recognition.

### Meaning

These findings suggest that to improve HCC surveillance attainment, barriers to surveillance receipt should be addressed.
