# Supplementary material for: Barriers to Surveillance for Hepatocellular Carcinoma in a Multicenter Cohort
Source: JAMA Netw Open. Author manuscript; Available in PMC 2022 Sep 7. (PMC9308050; doi:10.1001/jamanetworkopen.2022.23504)
Supplement: Supplementary Tables 1-4 — eTable 1. Factors Associated With Adequate Surveillance, Adjusted by Site eTable 2. Factors Associated With Curative-Intent Therapy of and Overall Survival, Adjusted By Site eTable 3. Factors Associated With Curative-Intent Therapy and Overall Survival, With Percentage of Time Under Surveillance eTable 4. Factors Associated With Curative-Intent Therapy and Overall Survival, With Percentage of Time Under Surveillance and Adjusted for Site [file NIHMS1826348-supplement-Supplementary_Tables_1-4.docx]

Supplemental File for: Barriers to Surveillance for Hepatocellular Carcinoma in a Multicenter Cohort

**Supplemental Table 1.** Predictor of adequate surveillance adjusted by site

| Variable | OR (95% CI) | p-value |
| --- | --- | --- |
| Age at HCC diagnosis in years | 1.02 (0.99 – 1.06) | 0.25 |
| Female gender (vs male) | 0.83 (0.43 – 1.56) | 0.58 |
| Race (ref White) | | |
| Black | 0.63 (0.29 – 1.29) | 0.22 |
| Other Race | 1.17 (0.39 – 3.21) | 0.77 |
| Hispanic ethnicity | 0.61 (0.16 – 2.25) | 0.46 |
| Body mass index (per unit) | 1.03 (0.99 – 1.07) | 0.15 |
| Etiology (ref viremic Hepatitis C) | | |
| Hepatitis C post SVR | 1.30 (0.66 – 2.53) | 0.44 |
| Alcohol related liver disease | 0.91 (0.40 – 1.99) | 0.82 |
| Non-alcohol associated fatty liver disease | 0.87 (0.36 – 2.02) | 0.76 |
| Hepatitis B | 3.87 (1.54 – 9.38) | **0.003** |
| Commercial/private insurance (ref: Medicare | 0.76 (0.43 – 1.30) | 0.32 |
| Child Pugh class B cirrhosis (versus Child Pugh class A) | 1.69 (1.01 – 2.82) | **0.04** |
| Cirrhosis diagnosed prior to HCC diagnosis (no vs yes) | 0.14 (0.02 – 0.48) | **0.009** |

Supplemental Table 2: Predictors of curative intent therapy of and overall survival adjusted by site; BMI: body mass index; SVR: sustained virological response; HCV: hepatitis C virus; ECOG: Eastern Cooperative Oncology Group; HCC: hepatocellular carcinoma

|  | *Curative Intent Therapy* | | *Overall Survival* | |
| --- | --- | --- | --- | --- |
| *Predictors* | *Odds Ratios (95% CI)* | *p-value* | *Hazard Ratios (95% CI)* | *p-value* |
| Age at diagnosis | 1.00 (0.97 – 1.03) | 0.791 | 1.01 (0.99 – 1.03) | 0.32 |
| Female gender | 1.23 (0.72 – 2.06) | 0.442 | 0.84 (0.60 – 1.17) | 0.30 |
| Race (ref White) |  |  |  |  |
| Black race | 1.94 (1.17 – 3.24) | **0.011** | 1.35 (0.99 – 1.84) | 0.06 |
| Other race | 0.97 (0.36 – 2.45) | 0.956 | 0.74 (0.43 – 1.29) | 0.29 |
| Hispanic ethnicity | 0.65 (0.21 – 2.07) | 0.457 | 1.00 (0.54 – 1.85) | 1.00 |
| BMI | 1.01 (0.97 – 1.04) | 0.653 | 0.97 (0.95 – 0.99) | **0.007** |
| Etiology of liver disease (ref viremic HCV) | | | | |
| Hepatitis C post SVR | 0.49 (0.26 – 0.91) | **0.026** | 0.89 (0.57 – 1.37) | 0.586 |
| Alcohol related liver disease | 0.95 (0.48 – 1.83) | 0.879 | 1.04 (0.70 – 1.57) | 0.834 |
| Non-alcohol associated fatty liver disease | 0.79 (0.38 – 1.63) | 0.529 | 1.57 (1.05 – 2.34) | **0.029** |
| Hepatitis B | 0.43 (0.15 – 1.11) | 0.103 | 1.38 (0.82 – 2.31) | 0.221 |
| Commercial insurance (vs Medicare) | 0.98 (0.63 – 1.52) | 0.916 | 0.97 (0.75 – 1.25) | 0.801 |
| ECOG 1 (vs 0) | 0.65 (0.36 – 1.13) | 0.137 | 1.11 (0.80 – 1.55) | 0.528 |
| ECOG ≥2 (vs 0) | 0.80 (0.33 – 1.80) | 0.597 | 2.44 (1.62 – 3.67) | **<0.001** |
| Child Pugh Class B (vs A) | 0.46 **(**0.29 – 0.72) | **0.001** | 1.76 **(**1.36 – 2.28) | **<0.001** |
| Cirrhosis not known prior to HCC diagnosis | 0.91 **(**0.51 – 1.60) | 0.754 | 1.08 **(**0.76 – 1.55) | 0.652 |
| Adequate surveillance | 1.73 (0.96 – 3.10) | 0.067 | 0.84 (0.56 – 1.28) | 0.425 |
| Intermittent surveillance | 0.48 (0.27 – 0.84) | **0.012** | 1.00 (0.74 – 1.37) | 0.981 |

**Supplemental Table 3:** Predictors of, curative intent therapy and overall survival with percent of time under surveillance. BMI: body mass index; SVR: sustained virological response; HCV: hepatitis C virus; NASH: nonalcoholic steatohepatitis; ECOG: Eastern Cooperative Oncology Group

|  | *Curative Intent Therapy* | | *Overall Survival* | |
| --- | --- | --- | --- | --- |
| *Predictors* | *Odds Ratios (95% CI)* | *p-value* | *Hazard Ratios (95% CI)* | *p-value* |
| Age at diagnosis | 0.99 (0.97 – 1.02) | 0.71 | 1.01 (0.99-1.03) | 0.30 |
| Female gender | 1.29 (0.79-2.09) | 0.31 | 0.84 (0.61-1.17) | 0.30 |
| Race (ref White) |  |  |  |  |
| Black race | 1.11 (0.71-1.74) | 0.65 | 1.37 (1.02-1.84) | **0.04** |
| Other race | 0.71 (0.26-1.77) | 0.48 | 0.74 (0.43-1.27) | 0.27 |
| Hispanic ethnicity | 0.37 (0.12-1.15) | 0.08 | 1.03 (0.56-1.89) | 0.91 |
| BMI | 1.01 (0.98-1.05) | 0.41 | 0.97 (0.95-0.99) | **0.007** |
| Etiology of liver disease (ref viremic HCV) | | | | |
| Hepatitis C post SVR | 0.49 (0.27-0.86) | **0.02** | 0.90 (0.58-1.38) | 0.62 |
| Alcohol related liver disease | 0.99 (0.53-1.83) | 0.98 | 1.06 (0.71-1.59) | 0.77 |
| Non-alcohol associated fatty liver disease | 0.97 (0.49-1.88) | 0.93 | 1.58 (1.06-2.35) | **0.03** |
| Hepatitis B | 0.37 (0.13-0.90) | **0.04** | 1.40 (0.84-2.31) | 0.19 |
| Commercial insurance (vs Medicare) | 0.95 (0.63-1.43) | 0.82 | 0.98 (0.76-1.27) | 0.90 |
| ECOG 1 (vs 0) | 1.26 (0.77-2.02) | 0.35 | 1.08 (0.80-1.47) | 0.61 |
| ECOG ≥2 (vs 0) | 1.22 (0.55-2.55) | 0.60 | 2.46 (1.64-3.67) | **<0.001** |
| Child Pugh Class B (vs A) | 0.41 (0.26-0.62) | **<0.001** | 1.81 (1.41-2.33) | **<0.001** |
| Cirrhosis not known prior to HCC diagnosis | 1.09 (0.62-1.89) | 0.76 | 1.02 (0.71-1.46) | 0.91 |
| Percentage of time under surveillance | 1.10 (1.03-1.18) | **0.005** | 0.97 (0.92-1.01) | 0.12 |

**Supplemental Table 4:** Predictors of, curative intent therapy and overall survival with percent of time under surveillance adjusted for site. BMI: body mass index; SVR: sustained virological response; HCV: hepatitis C virus; NASH: nonalcoholic steatohepatitis; ECOG: Eastern Cooperative Oncology Group

|  | *Curative Intent Therapy* | | *Overall Survival* | |
| --- | --- | --- | --- | --- |
| *Predictors* | *Odds Ratios (95% CI)* | *p-value* | *Hazard Ratios (95% CI)* | *p-value* |
| Age at diagnosis | 1.00 (0.97 – 1.03) | 0.85 | 1.01 (0.99-1.03) | 0.33 |
| Female gender | 1.20 (0.79-2.00) | 0.49 | 0.85 (0.61-1.18) | 0.33 |
| Race (ref White) |  |  |  |  |
| Black race | 1.77 (1.07-2.92) | **0.03** | 1.37 (1.00-1.87) | **0.05** |
| Other race | 1.00 (0.37-2.49) | 1.00 | 0.73 (0.42-1.27) | 0.26 |
| Hispanic ethnicity | 0.58 (0.19-1.83) | 0.35 | 1.05 (0.56-1.94) | 0.89 |
| BMI | 1.01 (0.97-1.04) | 0.69 | 0.97 (0.95-0.99) | **0.007** |
| Etiology of liver disease (ref viremic HCV) | | | | |
| Hepatitis C post SVR | 0.46 (0.25-0.84) | **0.01** | 0.90 (0.58-1.38) | 0.62 |
| Alcohol related liver disease | 0.92 (0.47-1.77) | 0.81 | 1.07 (0.71-1.60) | 0.75 |
| Non-alcohol associated fatty liver disease | 0.76 (0.37-1.54) | 0.45 | 1.58 (1.06-2.35) | **0.03** |
| Hepatitis B | 0.43 (0.15-1.10) | 0.10 | 1.39 (0.84-2.31) | 0.20 |
| Commercial insurance (vs Medicare) | 0.91 (0.59-1.41) | 0.68 | 0.98 (0.75-1.27) | 0.86 |
| ECOG 1 (vs 0) | 0.68 (0.38-1.17) | 0.17 | 1.11 (0.80-1.54) | 0.54 |
| ECOG ≥2 (vs 0) | 0.80 (0.34-1.78) | 0.61 | 2.43 (1.62-3.65) | **<0.001** |
| Child Pugh Class B (vs A) | 0.47 (0.30-0.74) | **0.001** | 1.79 (1.38-2.32) | **<0.001** |
| Cirrhosis not known prior to HCC diagnosis | 1.00 (0.55-1.77) | 0.99 | 1.02 (0.71-1.46) | 0.92 |
| Percentage of time under surveillance | 1.03 (0.95-1.11) | 0.51 | 0.97 (0.92-1.01) | 0.15 |
