# Supplementary material for: Barriers to Surveillance for Hepatocellular Carcinoma in a Multicenter Cohort
Source: JAMA Netw Open. 2022 Jul 22;5(7):e2223504. doi: 10.1001/jamanetworkopen.2022.23504 (PMC9308050; doi:10.1001/jamanetworkopen.2022.23504)
Supplement: Supplementary file 1 [file jamanetwopen-e2223504-s001.pdf]

Supplemental Online Content

Parikh ND, Tayob N, Al-Jarrah T, et al. Barriers to surveillance for hepatocellular carcinoma in a multicenter cohort. *JAMA Netw Open*. 2022;5(7):e2223504.
doi:10.1001/jamanetworkopen.2022.23504

eTable 1. Factors Associated With Adequate Surveillance, Adjusted by Site

eTable 2. Factors Associated With Curative-Intent Therapy of and Overall Survival, Adjusted By Site

eTable 3. Factors Associated With Curative-Intent Therapy and Overall Survival, With Percentage of Time Under Surveillance

eTable 4. Factors Associated With Curative-Intent Therapy and Overall Survival, With Percentage of Time Under Surveillance and Adjusted for Site

This supplemental material has been provided by the authors to give readers additional information about their work.

© 2022 Parikh ND et al. *JAMA Network Open.*

eTable 1. Factors Associated With Adequate Surveillance, Adjusted by Site

Variable	OR (95% CI)	p-value
Age at HCC diagnosis in years	1.02 (0.99 – 1.06)	0.25
Female gender (vs male)	0.83 (0.43 – 1.56)	0.58
Race (ref White)		
Black	0.63 (0.29 – 1.29)	0.22
Other Race	1.17 (0.39 – 3.21)	0.77
Hispanic ethnicity	0.61 (0.16 – 2.25)	0.46
Body mass index (per unit)	1.03 (0.99 – 1.07)	0.15
Etiology (ref viremic Hepatitis C)		
Hepatitis C post SVR	1.30 (0.66 – 2.53)	0.44
Alcohol related liver disease	0.91 (0.40 – 1.99)	0.82
Non-alcohol associated fatty liver disease	0.87 (0.36 – 2.02)	0.76
Hepatitis B	3.87 (1.54 – 9.38)	**0.003**
Commercial/private insurance (ref: Medicare	0.76 (0.43 – 1.30)	0.32
Child Pugh class B cirrhosis (versus Child Pugh class A)	1.69 (1.01 – 2.82)	**0.04**
Cirrhosis diagnosed prior to HCC diagnosis (no vs yes)	0.14 (0.02 – 0.48)	**0.009**

© 2022 Parikh ND et al. *JAMA Network Open.*

eTable 2. Factors Associated With Curative-Intent Therapy of and Overall Survival, Adjusted By Site

	Curative Intent Therapy		***Overall Survival***	
Predictors	*Odds Ratios (95% CI)*	*p-value*	*Hazard Ratios (95% CI)*	*p-value*
Age at diagnosis	1.00 (0.97 – 1.03)	0.791	1.01 (0.99 – 1.03)	0.32
Female gender	1.23 (0.72 – 2.06)	0.442	0.84 (0.60 – 1.17)	0.30
Race (ref White)				
Black race	1.94 (1.17 – 3.24)	**0.011**	1.35 (0.99 – 1.84)	0.06
Other race	0.97 (0.36 – 2.45)	0.956	0.74 (0.43 – 1.29)	0.29
Hispanic ethnicity	0.65 (0.21 – 2.07)	0.457	1.00 (0.54 – 1.85)	1.00
BMI	1.01 (0.97 – 1.04)	0.653	0.97 (0.95 – 0.99)	**0.007**
Etiology of liver disease (ref viremic HCV)				
Hepatitis C post SVR	0.49 (0.26 – 0.91)	**0.026**	0.89 (0.57 – 1.37)	0.586
Alcohol related liver disease	0.95 (0.48 – 1.83)	0.879	1.04 (0.70 – 1.57)	0.834
Non-alcohol associated fatty liver disease	0.79 (0.38 – 1.63)	0.529	1.57 (1.05 – 2.34)	**0.029**
Hepatitis B	0.43 (0.15 – 1.11)	0.103	1.38 (0.82 – 2.31)	0.221
Commercial insurance (vs Medicare)	0.98 (0.63 – 1.52)	0.916	0.97 (0.75 – 1.25)	0.801
ECOG 1 (vs 0)	0.65 (0.36 – 1.13)	0.137	1.11 (0.80 – 1.55)	0.528
ECOG ≥2 (vs 0)	0.80 (0.33 – 1.80)	0.597	2.44 (1.62 – 3.67)	**<0.001**
Child Pugh Class B (vs A)	0.46 (0.29 – 0.72)	**0.001**	1.76 (1.36 – 2.28)	**<0.001**
Cirrhosis not known prior to HCC diagnosis	0.91 (0.51 – 1.60)	0.754	1.08 (0.76 – 1.55)	0.652
Adequate surveillance	1.73 (0.96 – 3.10)	0.067	0.84 (0.56 – 1.28)	0.425
Intermittent surveillance	0.48 (0.27 – 0.84)	**0.012**	1.00 (0.74 – 1.37)	0.981

BMI: body mass index; SVR: sustained virological response; HCV: hepatitis C virus; ECOG: Eastern Cooperative Oncology Group; HCC: hepatocellular carcinoma

© 2022 Parikh ND et al. *JAMA Network Open.*

eTable 3. Factors Associated With Curative-Intent Therapy and Overall Survival, With Percentage of Time Under Surveillance

	Curative Intent Therapy		***Overall Survival***	
Predictors	*Odds Ratios (95% CI)*	*p-value*	*Hazard Ratios (95% CI)*	*p-value*
Age at diagnosis	0.99 (0.97 – 1.02)	0.71	1.01 (0.99-1.03)	0.30
Female gender	1.29 (0.79-2.09)	0.31	0.84 (0.61-1.17)	0.30
Race (ref White)				
Black race	1.11 (0.71-1.74)	0.65	1.37 (1.02-1.84)	**0.04**
Other race	0.71 (0.26-1.77)	0.48	0.74 (0.43-1.27)	0.27
Hispanic ethnicity	0.37 (0.12-1.15)	0.08	1.03 (0.56-1.89)	0.91
BMI	1.01 (0.98-1.05)	0.41	0.97 (0.95-0.99)	**0.007**
Etiology of liver disease (ref viremic HCV)				
Hepatitis C post SVR	0.49 (0.27-0.86)	**0.02**	0.90 (0.58-1.38)	0.62
Alcohol related liver disease	0.99 (0.53-1.83)	0.98	1.06 (0.71-1.59)	0.77
Non-alcohol associated fatty liver disease	0.97 (0.49-1.88)	0.93	1.58 (1.06-2.35)	**0.03**
Hepatitis B	0.37 (0.13-0.90)	**0.04**	1.40 (0.84-2.31)	0.19
Commercial insurance (vs Medicare)	0.95 (0.63-1.43)	0.82	0.98 (0.76-1.27)	0.90
ECOG 1 (vs 0)	1.26 (0.77-2.02)	0.35	1.08 (0.80-1.47)	0.61
ECOG ≥2 (vs 0)	1.22 (0.55-2.55)	0.60	2.46 (1.64-3.67)	**<0.001**
Child Pugh Class B (vs A)	0.41 (0.26-0.62)	**<0.001**	1.81 (1.41-2.33)	**<0.001**
Cirrhosis not known prior to HCC diagnosis	1.09 (0.62-1.89)	0.76	1.02 (0.71-1.46)	0.91
Percentage of time under surveillance	1.10 (1.03-1.18)	**0.005**	0.97 (0.92-1.01)	0.12

BMI: body mass index; SVR: sustained virological response; HCV: hepatitis C virus; NASH: nonalcoholic steatohepatitis; ECOG: Eastern Cooperative Oncology Group

© 2022 Parikh ND et al. *JAMA Network Open.*

eTable 4. Factors Associated With Curative-Intent Therapy and Overall Survival, With Percentage of Time Under Surveillance and Adjusted for Site

	Curative Intent Therapy		***Overall Survival***	
Predictors	*Odds Ratios (95% CI)*	*p-value*	*Hazard Ratios (95% CI)*	*p-value*
Age at diagnosis	1.00 (0.97 – 1.03)	0.85	1.01 (0.99-1.03)	0.33
Female gender	1.20 (0.79-2.00)	0.49	0.85 (0.61-1.18)	0.33
Race (ref White)				
Black race	1.77 (1.07-2.92)	**0.03**	1.37 (1.00-1.87)	**0.05**
Other race	1.00 (0.37-2.49)	1.00	0.73 (0.42-1.27)	0.26
Hispanic ethnicity	0.58 (0.19-1.83)	0.35	1.05 (0.56-1.94)	0.89
BMI	1.01 (0.97-1.04)	0.69	0.97 (0.95-0.99)	**0.007**
Etiology of liver disease (ref viremic HCV)				
Hepatitis C post SVR	0.46 (0.25-0.84)	**0.01**	0.90 (0.58-1.38)	0.62
Alcohol related liver disease	0.92 (0.47-1.77)	0.81	1.07 (0.71-1.60)	0.75
Non-alcohol associated fatty liver disease	0.76 (0.37-1.54)	0.45	1.58 (1.06-2.35)	**0.03**
Hepatitis B	0.43 (0.15-1.10)	0.10	1.39 (0.84-2.31)	0.20
Commercial insurance (vs Medicare)	0.91 (0.59-1.41)	0.68	0.98 (0.75-1.27)	0.86
ECOG 1 (vs 0)	0.68 (0.38-1.17)	0.17	1.11 (0.80-1.54)	0.54
ECOG ≥2 (vs 0)	0.80 (0.34-1.78)	0.61	2.43 (1.62-3.65)	**<0.001**
Child Pugh Class B (vs A)	0.47 (0.30-0.74)	**0.001**	1.79 (1.38-2.32)	**<0.001**
Cirrhosis not known prior to HCC diagnosis	1.00 (0.55-1.77)	0.99	1.02 (0.71-1.46)	0.92
Percentage of time under surveillance	1.03 (0.95-1.11)	0.51	0.97 (0.92-1.01)	0.15

BMI: body mass index; SVR: sustained virological response; HCV: hepatitis C virus; NASH: nonalcoholic steatohepatitis; ECOG: Eastern Cooperative Oncology Group

© 2022 Parikh ND et al. *JAMA Network Open.*
